# CCR4 as a Therapeutic Target for Cancer Immunotherapy

**DOI:** 10.3390/cancers13215542

**Published:** 2021-11-04

**Authors:** Osamu Yoshie

**Affiliations:** 1 Health and Kampo Institute, Sendai 981-3205, Japan; qqud7p89k@siren.ocn.ne.jp; 2 Kindai University, Osaka 577-8502, Japan; 3 Aoinosono-Sendai Izumi Long-Term Health Care Facility, Sendai 981-3126, Japan

**Keywords:** chemokine, chemokine receptor, CCR4, T cell subset, HTLV-1, ATLL, CTLL, HAM/TSP, Mogamulizumab, immune check point inhibitor

## Abstract

**Simple Summary:**

CCR4 is a chemokine receptor selectively expressed on normal T cell subsets such as type 2 helper T cells, skin-homing T cells and regulatory T cells, and on skin-associated T cell malignancies such as adult T cell leukemia/lymphoma (ATLL), which is etiologically associated with human T lymphocyte virus type 1 (HTLV-1), and cutaneous T cell lymphomas (CTCLs). Mogamulizumab is a fully humanized and glyco-engineered monoclonal anti-CCR4 antibody used for the treatment of refractory/relapsed ATLL and CTCLs, often resulting in complete remission. The clinical applications of Mogamulizumab are now being extended to solid tumors, exploring the therapeutic effect of regulatory T cell depletion. This review overviews the expression of CCR4 in various T cell subsets, HTLV-1-infected T cells, ATLL and CTCLs, and the clinical applications of Mogamulizumab.

**Abstract:**

CCR4 is a chemokine receptor mainly expressed by T cells. It is the receptor for two CC chemokine ligands, CCL17 and CCL22. Originally, the expression of CCR4 was described as highly selective for helper T type 2 (Th2) cells. Later, its expression was extended to other T cell subsets such as regulatory T (Treg) cells and Th17 cells. CCR4 has long been regarded as a potential therapeutic target for allergic diseases such as atopic dermatitis and bronchial asthma. Furthermore, the findings showing that CCR4 is strongly expressed by T cell malignancies such as adult T cell leukemia/lymphoma (ATLL) and cutaneous T cell lymphomas (CTCLs) have led to the development and clinical application of the fully humanized and glyco-engineered monoclonal anti-CCR4 Mogamulizumab in refractory/relapsed ATLL and CTCLs with remarkable successes. However, Mogamulizumab often induces severe adverse events in the skin possibly because of its efficient depletion of Treg cells. In particular, treatment with Mogamulizumab prior to allogenic hematopoietic stem cell transplantation (allo-HSCT), the only curative option of these T cell malignancies, often leads to severe glucocorticoid-refractory graft-versus-host diseases. The efficient depletion of Treg cells by Mogamulizumab has also led to its clinical trials in advanced solid tumors singly or in combination with immune checkpoint inhibitors. The main focus of this review is CCR4; its expression on normal and malignant T cells and its significance as a therapeutic target in cancer immunotherapy.

## 1. Introduction

In 2014, Kouji Matsushima and I wrote a review entitled “CCR4 and its ligands: from bench to bedside” in the International Journal of Immunology [1]. The review was intended to cover the functional association of CCR4 with various T subsets and also to introduce the exciting success and subsequent approval of Mogamulizumab, a humanized and glyco-engineered monoclonal anti-CCR4, in the treatment of refractory/relapsed adult T-cell leukemia/lymphoma (ATLL) in Japan. Subsequently, Mogamulizumab has been approved in the US for the treatment of cutaneous T cell lymphomas (CTCLs), namely, mycosis fungoides (MF) and Sézary syndrome (SS), after at least one prior systemic treatment. The potential clinical application of Mogamulizumab is now extended to solid tumors because of its efficient depletion of regulatory T (Treg) cells.

In this review, I first overview the chemokine superfamily and the differentiation of naïve T cells into various functional T cell subsets accompanied by characteristic expressions of selective chemokine receptors. I then describe the human oncogenic retrovirus HTLV-1 (human T lymphocyte virus type 1) and its etiologically associated diseases, adult T-cell leukemia/lymphoma (ATLL) and HTLV-1-associated myelopathy/tropical spastic paraparesis (HAM/TSP), with particular attention on the expression of CCR4 on HTLV-1-infected T cells and malignant T cells. Then, I describe the development of Mogamulizumab, its successful clinical applications to ATLL and cutaneous T cell lymphomas (CTCLs), and its frequent adverse events apparently related to the efficient depletion of Treg cells. Next, I briefly overview the application of immune checkpoint inhibitors to various cancers including ATLL and CTCLs. Finally, I describe the recent clinical trials of Mogamulizumab in solid tumors, alone or in combination with immune checkpoint inhibitors. Overall, this review intends to update the various topics related to CCR4 and the clinical applications of Mogamulizumab since our 2014 review [1].

## 2. Overview on the Chemokine Superfamily and T Cell Subsets

### 2.1. The Chemokine Superfamily

**Figure 1:**
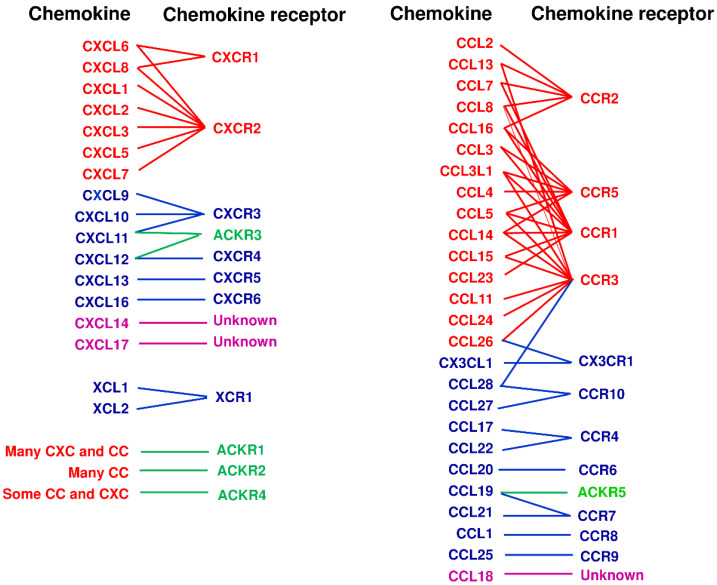
The chemokine superfamily. Inflammatory chemokines and their receptors are indicated in red. Immune chemokines (homeostatic/dual chemokines) are indicated in blue. Atypical chemokine receptors are indicated in green. Chemokines with unknown receptors are indicated in purple. The lines indicate well-established agonistic interactions. Antagonistic or indirect interactions are not shown.

Chemokines are a group of small secreted proteins and two transmembrane molecules that are primarily important for migration and tissue infiltration of leukocytes and lymphocytes [2,3,4,5]. Chemokines are also involved in migration, survival, and tissue localization of various types of cells including cancer cells [2,3,4,5]. Chemokine molecules have four well-conserved cysteine residues and were originally divided into the CC and CXC subfamilies according to the motif of two N-terminal cysteine residues; they are adjacent in the CC subfamily and separated by a single amino acid in the CXC subfamily. Subsequently two new types of chemokine were identified, representing the (X)C subfamily that lacks the first and third cysteine residues, and the CX_3_C subfamily that has three amino acids between the two N-terminal cysteine residues [2]. In humans, close to 50 chemokine molecules are now known [1,3] (Figure 1). Chemokine receptors are also a subfamily of the superfamily of seven-transmembrane domain G protein-coupled receptors (GPCRs). In humans, there are eighteen chemotactic receptors and five non-chemotactic/atypical scavenging receptors [1,3] (Figure 1). The chemokines and their receptors are now indicated by their systematic names, consisting of the subfamily CC, CXC, (X)C or CX_3_C followed by L for ligand or R for receptor and a number allocated principally by the order of discovery [2]. Atypical chemokine receptors are indicated by the abbreviation ACKR and numbers.

Functionally, chemokines are often classified into inflammatory and homeostatic chemokines according to their mode of expression and biological functions. However, such classifications are oversimplistic; even inflammatory chemokines may have some homeostatic roles whereas homeostatic chemokines often have inflammatory roles. Thus, some chemokines are now called dual chemokines because of their homeostatic and inflammatory functions [3]. Originally, however, I called the chemokines that mainly target lymphocytes as “immune chemokines” because of not only their main roles in lymphocyte migration but also their more restricted ligand–receptor relationships and their evolutionary differences from the inflammatory chemokines as evidenced from their genes being mapped at loci different from the major gene clusters of inflammatory chemokines that are located in chromosome 4 for CXC chemokines and chromosome 17 for CC chemokines in humans [6]. Thus, personally, I prefer to use the term ‘immune chemokines’ for most of the homeostatic and dual chemokines (Table 1). At any rate, typical inflammatory chemokines and their receptors have highly redundant ligand–receptor relationships, while so-called “immune chemokines” tend to have more restricted ligand–receptor relationships [2,3] (Figure 1). However, even if there are multiple ligands for a receptor and multiple receptors for a ligand by in vitro assays, their respective functions in vivo may be different through possible differences in induction signals, producing cells, and/or biased signaling events after receptor binding [7]. Indeed, among the so-called “immune chemokines”, the axes of CCR4, CCR7 and CXCR3 have two or three ligands for a single receptor (Table 1), but the ligands have biased intracellular signaling functions and complementary roles in the tissue microenvironment [1,7,8].

### 2.2. Identification of CCR4

CCR4 was initially reported as a novel chemokine receptor for the three major inflammatory chemokines MCP-1/CCL2, MIP-1α/CCL3 and RANTES/CCL5 [9]. However, we re-identified CCR4 as a highly specific receptor for two newly identified and closely related CC chemokines [10,11]: TARC/CCL17, a novel T-cell attracting CC chemokine [12], and MDC/STCP-1/CCL22, another novel CC chemokine derived from macrophages [13,14]. On the other hand, we confirmed that CCR4 had no receptor functions for CCL2, CCL3 or CCL5 even when using highly sensitive binding, chemotactic or calcium mobilization assays [10]. We have further demonstrated that the CCL17 and CCL22 genes are closely mapped to the human chromosome 16q13 together with that of CX_3_CL1 [11,15,16], suggesting that CCL17 and CCL22 were generated from a common ancestor by gene duplication and thus share the same receptor CCR4. Mouse Ccl17, Ccl22, and Ccr4 were also cloned and characterized. Their functional properties are highly consistent with those of human counterparts [17,18,19,20]. Mouse Ccl17 and Ccl22 genes are also closely mapped to the mouse chromosome 8qC5 together with that of Cx_3_cl1 [21].

We and others have also demonstrated that CCL17 and CCL22 as well as their mouse counterparts are not equivalent agonists for CCR4 or Ccr4. CCL22 is dominant over CCL17 in terms of CCR4 desensitization: namely, ligand-induced internalization of CCR4 [11,20,22]. Thus, while pretreatment with CCL22 desensitizes CCR4 to subsequent stimulation with CCL17, pretreatment with CCL17 does not efficiently desensitize CCR4 to CCL22 [11,20,22]. This is considered to be relevant to their respective roles in T cell trafficking to the skin [23]. CCL17 is expressed by dermal endothelial cells and promotes extravasation of T cells via CCR4 [24]. Since CCL17 does not efficiently desensitize CCR4, CCR4-expressing T cells are still able to migrate within the skin tissue toward CCL22 that is mainly produced by dermal dendritic (DC) cells [23]. On the other hand, CCL22 but not CCL17 has the N-terminal Gly_1_-Pro_2_ (the X_1_P_2_ motif), the cleavage site for CD26/dipeptidyl peptidase 4 (DPP4), which is expressed on various cells including endothelial cells. Since the cleavage of the N-terminus by DPP4 makes CCL22 poorly active on CCR4, it prevents CCL22 acting on CCR4 prior to CCL17 on endothelial cells. The cleavage of CCL22 by DPP4 was, however, found to proceed beyond Gly_1_-Pro_2_ to Tyr_3_-Gly_4_, resulting in the generation of CCL22 (5–69) [25]. It was further reported that intact as well as truncated CCL22 was equally chemotactic for monocytes, suggesting, though not yet proven, the presence of an alternative receptor for CCL22 [25]. It has also been reported that CCR4 exists in at least two distinct conformational populations, of which a major population is activated by both CCL17 and CCL22, while a minor population is activated only by CCL22 [22].

### 2.3. T Cell Subsets

After exit from the thymus, naïve T cells are known to recirculate between the blood and secondary lymphoid organs (SLOs). On the other hand, immature DCs present in peripheral tissues start maturation upon antigenic stimuli and migrate to SLOs via afferent lymphatics. In SLOs, naïve T cells meet antigen-loaded mature DCs and scan antigenic peptides presented in association with major histocompatibility complex (MHC) molecules. These migratory and interactive processes of naïve T cells and DCs are crucially dependent on CCR7 and its two ligands CCL19 and CCL22 [26,27,28]. If not stimulated by antigenic peptides, naïve T cells exit SLOs via efferent lymphatics by using sphingosine-1-phosphate (S1P) and its receptor S1P-R1 [29,30]. If stimulated by cognate antigenic peptide-MHC complexes and CD80/CD86 co-stimulatory molecules via CD28, naïve T cells proliferate and differentiate into various effector T cell subsets in accordance with local cytokine milieu and imprinting conditions. Accordingly, effector CD4^+^ T cells are composed of various functional subsets such as Th1, Th2, Th17, Th22, Tfh, and Treg (Table 2), although such functional differentiations are often flexible and can be overlapping [31,32]. It is now known that during T cell activation, local cytokine milieu drives the expression of distinct master transcription factors in activated T cells that drive Th phenotype differentiation and production of signature cytokines. The respective master regulators are T-box 21 (T-bet) for Th1, GATA binding protein 3 (GATA3) for Th2, RAR-related orphan receptor γ (ROR-γ) for Th17, Aryl hydrocarbon receptor (AHR) for Th22, B cell lymphoma 6 protein (Bcl-6) for Tfh, and Forkhead box P3 (FoxP3) for Treg [33]. Each T cell subset is primarily characterized by production of signature cytokines and also by expression of selective chemokine receptors (Table 2).

Antigen-primed T cells also differentiate into long-lived memory T cells, which are now divided into two major subsets: central memory T (Tcm) cells and effector memory T (Tem) cells. Tcm cells express CCR7 and L-selectin and preferentially traffic to SLOs whereas Tem cells are devoid of the expression of CCR7 or L-selectin and broadly migrate between peripheral tissues, the blood, and the spleen, using various chemokine receptors and adhesion molecules [34,35].

### 2.4. T Cell Subsets and Chemokine Receptors

Th1 cells and Th2 cells are the originally described subsets of T helper cells [36,37]. Th1 cells are involved in cellular immunity, produce type 1 cytokines such as IFN-γ and TNF-α, and are important effector cells against intracellular pathogens such as mycobacteria and viruses, whereas Th2 cells are involved in humoral immunity, produce type 2 cytokines such as IL-4, IL-5 and IL-13, and are important effector cells against extracellular pathogens such as conventional bacteria and parasites [36,37]. This dichotomy of effector function is also accompanied by distinct migratory properties. We and others have originally demonstrated that Th2 cells express CCR4 whereas Th1 cells express CXCR3 [1,38,39]. Consistently, we and others have also shown that the CCR4 ligands CCL17 and CCL22 are highly expressed in allergic diseases such as atopic dermatitis and bronchial asthma [1]. In particular, we and others have shown that the levels of CCL17 and CCL22 in the blood are highly elevated in patients with atopic dermatitis and correlate well with disease severity and treatment response [1,40,41]. Accordingly, CCL17/TARC was approved by the Japanese Pharmaceutical and Medical Devices Agency (PMDA) as a clinical test reagent in 2008 and is now regarded as an excellent blood biomarker for disease severity and therapeutic response in atopic dermatitis [1,42]. On the other hand, the CXCR3 ligands, MIG/CXCL9, IP-10/CXCL10 and I-Tac/CXCL11, are commonly induced by type 1 and type 2 interferons (IFNs) in accordance with the roles of CXCR3-expressing cells such as Th1 cells and effector CD8^+^ T cells in cellular immunity [7]. Currently, it is the consensus understanding that Th1 cells mainly express CXCR3 and CCR5, whereas Th2 cells express CCR4, CCR8, and, to a lesser extent, CCR3 [43] (Table 2).

Since the original description of Th1 and Th2, the diversity of T cell subsets has been greatly expanded (Table 2). Th17 cells produce IL-17A, IL-17F and IL-22 as signature cytokines, are important for clearance of extracellular bacterial and fungal infections, and are also involved in inflammatory and autoimmune diseases [44,45]. Th17 cells have been shown to express CCR6 and CCR4 [46]. Th22 cells secrete IL-22, TNF-α and IL-13, but not IL-17 or IFN-γ, co-express CCR6, CCR4 and CCR10, and are involved in skin homeostasis and inflammation [47,48,49]. Tfh cells are present in the germinal center of SLOs, express CXCR5, and also produce its ligand CXCL13, which attracts CXCR5-expressing B cells to germinal centers. Tfh cells promote antibody production, immunoglobulin class switching, and somatic hypermutation of B cells that have responded to T-dependent antigens [50]. Treg cells are known to play a central role in maintaining immunological tolerance to self-antigens and in curtailing excessive immune responses deleterious to the host [51]. CD4^+^CD25^+^cytotoxic T lymphocyte antigen (CTLA)-4^+^ Treg cells were originally reported to express CCR4 and CCR8 [52]. It has also been reported that CD4^+^CD25^high^Foxp3^+^ Treg cells in human peripheral blood express CCR4 at high levels and the majority of them also express CCR6 and cutaneous lymphocyte antigen (CLA), the phenotype of skin-homing T cells [53].

### 2.5. Tissue Imprinting and Chemokine Receptors

Another important functional differentiation of T cells is determined by tissue-specific imprinting, which is promoted by tissue-localized DCs and tissue-specific environmental cues [54]. DCs in the gut imprint T cells to express α4β7 and CCR9, which enable their entry into the small intestine in response to MAdCAM-1 and TECK/CCL25, respectively [55,56,57]. It has been shown that retinoic acid, the vitamin A metabolite, produced by gut DCs induces the expression of α4β7 and CCR9 in activated T cells [58]. Similarly, skin-derived DCs imprint T cells to express CLA and CCR10, which allow their skin homing via cutaneous P- and E-selectins expressed on skin microvasculature endothelium and CTACK/CCL27 secreted by epidermal keratinocytes, respectively [59,60]. The active form of vitamin D_3_ produced by skin DCs has been shown to induce T cells to express CCR10 [61,62]. In mice, CCR10 has been shown to be preferentially expressed on memory-like skin-resident T cells but not on most skin-infiltrating effector T cells during inflammation, suggesting that CCR10-expressing skin-resident T cells are mainly involved in skin homeostasis [63].

The expression of CCR8 is also a characteristic for long-lived memory T cells in human skin [64]. Epidermal keratinocytes produce skin-specific factors, including 1α,25-dihydroxyvitamin D_3_, the vitamin D_3_ metabolite, and prostaglandin E_2_ (PGE_2_), to induce the expression of CCR8 during naïve T cell activation [65]. In a mouse study, CCR8-deficient mice developed severe contact hypersensitivity through expansion of Th1 and Th17 cell populations [66]. Furthermore, skin DCs were shown to migrate readily to the draining lymph nodes upon antigenic sensitization. Thus, CCR8-expressing T cells may negatively regulate the migration of DCs from the skin to the draining lymph nodes [66].

In another mouse study, lung DCs were found to imprint T cells for lung homing, since T cells activated by lung DCs migrated more efficiently into the lungs in homeostasis and in response to an inhaled antigen compared with T cells activated by DCs from other organs [67]. Lung DCs were also found to induce CCR4 expression in activated T cells. Furthermore, lung DC-activated CCR4-deficient T cells failed to traffic into the lungs as efficiently as lung DC-activated CCR4-sufficient T cells. Thus, lung DCs may imprint T cells for lung homing in part by inducing CCR4 expression [67].

## 3. HTLV-1, ATLL and HAM/TSP

### 3.1. HTLV-1, a Human Oncogenic Retrovirus

Human T-cell leukemia virus type 1 (HTLV-1) was discovered as the first human retrovirus and shown to be etiologically associated with a highly aggressive mature CD4^+^ T cell malignancy, adult T-cell leukemia/lymphoma (ATLL) [68,69]. The virus was also etiologically associated with HTLV-1-associated myelopathy/tropical spastic paralysis (HAM/TSP), a chronic disease characterized by progressive spasticity, bladder disfunction, and sensory deficits [70,71]. HTLV-1 is endemic in certain isolated areas of the world such as Southwest Japan, sub-Saharan Africa, Caribbean islands, Australia, Iran, and South America [72,73]. The number of people infected with HTLV-1 is estimated to be 5 to 10 million worldwide. Importantly, HTLV-1 is poorly infectious as cell-free virions, and its transmission from person to person requires a direct cell-to-cell contact between HTLV-1-infected cells and target cells [74,75]. Thus, live HTLV-1-infected cells need to be transferred from a virus carrier to an uninfected person. Accordingly, the main routes of HTLV-1 infection are mostly familial: vertical transmission from mother to child via breast feeding and sexual transmission primarily from male to female. The modern third route is blood transfusion or intravenous drug use [75]. Within the body, the viral transmission occurs to CD4^+^ T cells and, to a lesser degree, to cells such as CD8^+^ T cells through a cell-to-cell contact [74,76,77]. However, it has been shown that DCs can be uniquely infected by cell-free virions and may function as an important viral reservoir in the body [75,78]. The cell-dependent mode of transmission is certainly the main reason for the genetic stability of proviral HTLV-1 and its endemicity in certain remote areas of the world where indigenous peoples carrying HTLV-1 have long maintained isolated communities. This contrasts to the pandemic infection and genetic instability of human immunodeficiency virus type 1 (HIV-1), another human retrovirus discovered shortly after HTLV-1, which infects people by cell-free virions [79]. However, it should also be noted that HTLV-1 infection is now spreading to non-endemic areas through increased mobilization and mixing of people worldwide [80,81].

HTLV-1 encodes a potent viral transactivator Tax, which promotes viral gene expression via the long terminal repeat (LTR) and also potently activates signaling pathways such as nuclear factor NF-κB to induce various cellular genes involved in T cell proliferation and survival, resulting in rapid expansion of infected T cells in the body [82,83]. However, Tax is also highly immunogenic and provides a good target for host CD8^+^ T cell responses [84]. Accordingly, HTLV-1-infected T cells expressing Tax are efficiently eliminated by Tax-specific cytotoxic T cells, and Tax is almost completely shut down in surviving HTLV-1-infected T cells by mechanisms such as abortive mutations in the Tax gene or DNA hypermethylation in the 5′ LTR [85]. Nevertheless, the impact of Tax on the expression of cellular genes is maintained in infected cells even after the shutdown of its expression through genetic and epigenetic alterations [83,85]. Furthermore, HTLV-1 has another important transcription factor HTLV-1 bZIP factor (HBZ), which is encoded on the anti-sense strand [86]. HBZ is not immunogenic, constitutively expressed in HTLV-1-infected T cells, and has been shown to promote proliferation and survival of HTLV-1-infected T cells [86]. Thus, both Tax and HBZ are now regarded as the major HTLV-1 oncogenes [87].

### 3.2. ATLL and HAM/TSP

ATLL is a malignancy of mostly CD4^+^ mature T cells that occurs in about 5% of HTLV-1 carriers mostly infected by breast feeding and after decades of latency [81,88]. There are four clinical types in ATLL: acute, lymphoma, smoldering, and chronic. The chronic type is now further subdivided into the favorable and unfavorable types. Acute, lymphoma and unfavorable chronic types of ATLL are defined as aggressive, whereas smoldering and favorable chronic types are defined as indolent [89]. For indolent ATLL, an antiretroviral treatment with azidothymidine (AZT) and IFN-α may be temporarily effective [90]. On the other hand, aggressive ATLL is highly resistant to the current chemotherapeutic regimen with an average survival time of less than a year [81,88]. Allogenic hematopoietic stem cell transplantation (allo-HSCT) is the only curative treatment for aggressive ATLL [91]. One of the advantages of allo-HSCT is the induction of graft-versus-leukemia responses in the recipient [91,92]. However, it is not feasible for the majority of patients because of the poor response of ATLL to chemotherapy and of the advanced age of most patients. Furthermore, because of high morbidity and mortality rates, the success rate of allo-HSCT is only one-third of recipients [91].

In addition to ATLL, HTLV-1 infection is also etiologically associated with several autoimmune-like inflammatory diseases, the most important being HAM/TSP, which is a demyelinating disease involving primarily the spinal cord and develops in about 4% of HTLV-1 carriers [93,94]. It is characterized by chronic progressive spasticity and weakness, bladder disfunction, and increasing sensory deficits of the lower extremities [93,94]. In contrast to ATLL, HAM/TSP tends to develop in HTLV-1 carriers infected in adulthood through sexual transmission or contaminated blood [93,94]. Corticosteroids constitute the current major treatments of HAM/TSP but only with partial effectiveness [94].

## 4. Expression of CCR4 in ATLL, HAM/TSP, and CTCLs

### 4.1. Preferential Transmission of HTLV-1 to CCR4-Expressing T Cells

**Figure 2:**
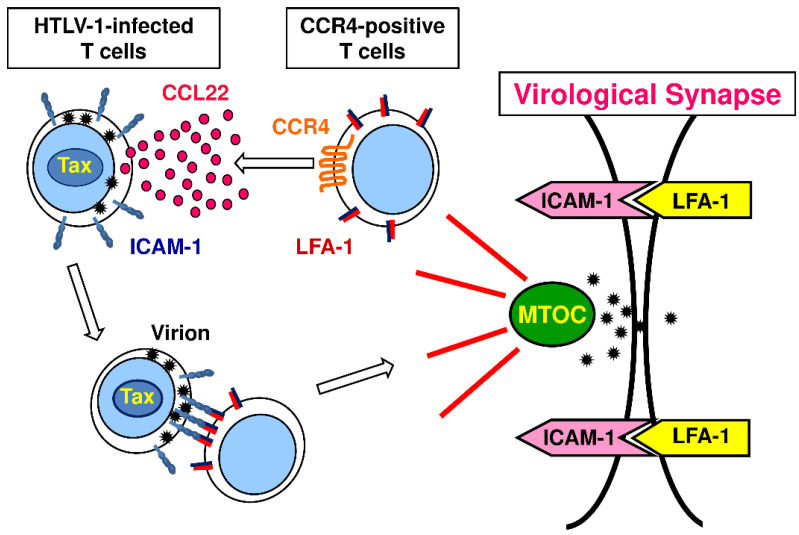
Preferential transmission of HTLV-1 virions from HTLV-1-infected CD4^+^ T cells to CCR4-expressing CD4^+^ T cells via the virological synapse. HTLV-1 Tax induces ICAM-1 upregulation and CCL22 production in HTLV-1-infected T cells. CCL22 attracts CCR4-expressing T cells to HTLV-1-infected T cells and activates LFA-1. Binding of activated LFA-1 to ICAM-1 induces the formation of the virological synapse. MTOC, microtubule organizing center.

The CCR4 gene is not inducible by Tax [95]. Nevertheless, HTLV-1-infected T cells mostly express CCR4 [95]. Thus, although HTLV-1 is capable of infecting various types of cells including CD4^+^ T cells, CD8^+^ T cells, monocytes, and DCs [77], CCR4^+^CD4^+^ T cells appear to be the preferential target cells for viral transmission and/or persistency [95]. For the preferential transmission of HTLV-1 to CCR4^+^CD4^+^ T cells, the following scenario is now considered (Figure 2). First, we and others have shown that intercellular adhesion molecule 1 (ICAM-1) is strongly upregulated in HTLV-1-infected T cells by Tax [96,97]. Second, we and others have also shown that CCL22, one of the CCR4 ligands, is potently induced in HTLV-1-infected T cells by Tax [76,98]. Accordingly, HTLV-1-infected T cells express ICAM-1 at high levels and abundantly produce CCL22. Thus, HTLV-1-infected T cells preferentially attract CCR4-expressing T cells via CCL22. Furthermore, as chemokines are generally known to activate integrins, CCL22 activates lymphocyte function-associated antigen 1 (LFA-1), an integrin, expressed on attracted CCR4-expressing T cells to promote its firm binding to ICAM-1 expressed on HTLV-1-infected T cells. This triggers reorientation of the cytoskeleton and polarization of the microtubule organizing center (MTOC) towards the cell–cell junction in HTLV-1-infected T cells, leading to the formation of a virological synapse, “the virus-induced specialized area of cell–cell contact that promotes the directed transmission of the virus between cells” [76]. The virological synapse thus permits the efficient transmission of HTLV-1 virions from HTLV-1-infected T cells to uninfected CCR4^+^CD4^+^ T cells [76,98]. In addition, CCL22 production by HTLV-1-infected T cells may also help their survival by attracting Treg cells to generate an immunosuppressive microenvironment [99].

### 4.2. CCR4 Expression in ATLL

Since the chemokine receptors are useful surface makers for various T cell subsets (Table 2), they should be also useful surface markers for their neoplastic counterparts. Indeed, we and others have shown that CCR4 is highly expressed in ATLL [95,100]. This may indicate that ATLL is predominantly derived from CCR4-expressing T cells such as Th2 cells and Treg cells [95,101,102]. CCR4 expression may also explain the frequent skin infiltration of ATLL [95,100,103]. Frequent co-expression of CCR10 in ATLL may further promote skin infiltration [104]. Furthermore, CCR7 is often highly expressed on ATLL and positively correlates with the involvement of lymphoid organs [105].

**Figure 3:**
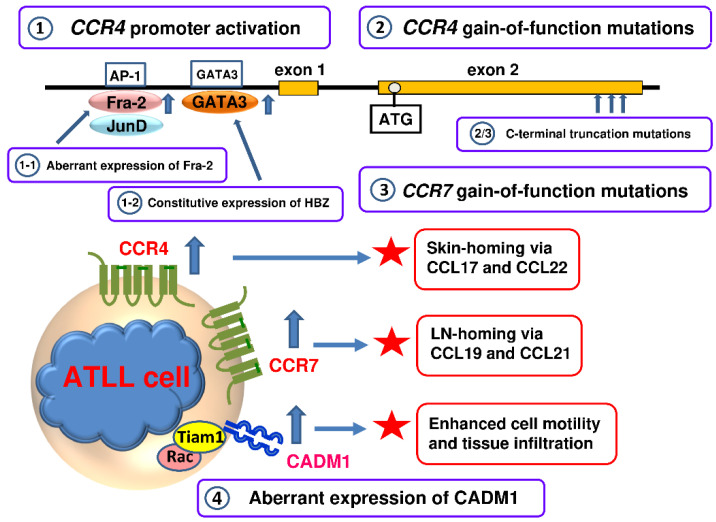
Multiple mechanisms are involved in the augmented expression of CCR4, CCR7 and CAMD1 in ATLL. CADM1, cell adhesion molecule 1.

We have also observed that ATLL cells in the blood of patients often express CCR4 at levels much higher than normal CCR4-expressing CD4^+^ T cells and also migrate toward CCL17 and CCL22 much more vigorously than normal CCR4-expressing CD4^+^ T cells [95,106]. This suggested that some potent transcription factors that were constitutively active in ATLL and thus likely to be oncogenic were also responsible for the strong expression of CCR4. We therefore performed CCR4 promoter analysis in ATLL cells and found that the GATA3 and AP-1 sites in the proximal region of the CCR4 promoter were mostly responsible for the expression of CCR4 in ATLL cells [107]. The AP-1 family transcription factors are well known regulators of many biological processes such as proliferation, differentiation, survival, and apoptosis [108]. Furthermore, the deregulated expression of AP-1 transcription factors is implicated in oncogenesis of various lymphomas including ATLL [109,110]. We therefore focused on the expression of the AP-1 family members in ATLL [107]. We have shown that Fos-related antigen 2 (Fra-2) is highly expressed in ATLL and, in association with another AP-1 family member JunD, strongly activates the CCR4 promoter [107] (Figure 3). We have further shown that both Fra-2 and JunD are necessary for proliferation of ATLL cells and involved in the induction of various growth-related genes. One of the induced genes is a member of the Sry-related high-mobility group box family of transcription factors SOX4, which is frequently overexpressed in various malignancies [107]. We have further demonstrated that SOX4 is also involved in the proliferation of ATLL and induces histone deacetylase 8 (HDAC8) among other target genes [111]. A recent study has also reported strong expression and diagnostic utility of SOX4 in ATLL [112]. It has been further reported that a pan-histone deacetylase inhibitor Vorinostat as well as a class I selective histone deacetylase inhibitor Romidepsin downregulates CCR4 expression in ATLL [113]. By using the short-interfering RNA technique, HDAC2 has been shown to promote CCR4 expression in ATLL [113]. In addition, HBZ, which is constitutively expressed in ATLL, has also been shown to upregulate CCR4 expression by inducing GATA3 [114] (Figure 3).

Besides the transcriptional activation of the CCR4 promoter in ATLL by transcription factors such as Fra-2/JunD and HBZ-induced GATA-3 [107,114], recent studies have further revealed another striking mechanism for the enhanced expression of CCR4 in ATLL. First, an extensive transcriptome study found nonsense or frameshift mutations in the CCR4 gene in 14 out of 53 (26%) ATLL samples. Furthermore, these mutations were commonly gain-of-function mutations, resulting in truncation of the carboxy terminus of CCR4 at C329, Q330 or Y331. Since the C-terminal intracellular domain of G protein-coupled receptors is essential for the ligand-induced internalization and desensitization, CCR4 with a truncated C-terminus remains on the cell surface at high levels by resisting ligand-induced internalization and thus mediates vigorous cell migration toward CCL17 and CCL22 [115]. Second, a highly integrated molecular study involving whole-genome, exome, transcriptome and targeted resequencing as well as array-based copy number and methylation analyses has been conducted in Japan using a total of 426 ATLL cases [116]. This monumental study has also confirmed frequent gain-of-function mutations in the CCR4 gene (29%). Furthermore, this study has revealed similar gain-of-function mutations in the CCR7 gene (11%). The latter is consistent with the reported CCR7 expression at high levels in a fraction of ATLL patients [105]. Other identified gene alterations found in this study significantly overlap with the HTLV-1 Tax interactome and are highly enriched for T cell receptor/NF-κB signaling and other T cell-related pathways [116]. Of note, North American ATLL patients who are mostly of Caribbean descent are characterized by a relatively younger age and worse prognosis than ATLL patients in Japan [117]. A targeted exon sequencing study of North American ATLL patients (*n* = 30) has revealed that the mutation frequency in epigenetic and histone modifying genes is significantly higher and the mutation frequency in JAK/STAT and T cell/NF-κB pathway genes is significantly lower compared with Japanese ATLL patients [117]. Thus, North American ATLL has a distinct genomic landscape with aberrant hypermethylation and inactivation of tumor suppressor genes playing a dominant role in the pathogenic mechanisms.

Collectively, multiple mechanisms have been found to be involved in the enhanced expression of CCR4 in ATLL (Figure 3). The expression of CCR4 and that of CCR7 account for the frequent infiltration of the skin and SLOs in ATLL, respectively (Figure 3). Furthermore, the frequent gain-of-function mutations in CCR4 and CCR7 in ATLL strongly suggest selective advantages of constitutive expression of CCR4 and CCR7 in ATLL with respect to cell proliferation and/or survival in the tissue microenvironment [118,119,120]. Yet another important surface molecule that is overexpressed by ATLL is cell adhesion molecule 1 (CADM1)/tumor suppressor in lung cancer 1 (TSLC1) [121,122]. CADM1 is not expressed on normal T cells but is highly expressed on HTLV-1-infected T cells and thus provides the best single surface marker of HTLV-1-infected T cells [122,123]. Furthermore, CADM1 expression tends to be higher in carriers in disease progression and in HAM/TSP patients whose symptoms are worsening [124,125]. CADM1 has been shown to interact with T-lymphoma invasion and metastasis 1 (Tiam1) via the cytoplasmic domains and induces the formation of lamellipodia through Rac activation, thus enhancing migration and tissue infiltration of HTLV-1-infected T cells and ATLL cells [126] (Figure 3).

### 4.3. Expression of CCR4 in CTCLs

Cutaneous T-cell lymphomas (CTCLs) are a heterogenous group of non-Hodgkin lymphomas derived from skin-homing T cells. MF and SS are the most common types of CTCLs [127,128,129]. MF is a disease of indolent clinical course with slow progression, proceeding from patches, to more infiltrated plaques, and eventually to tumors. Histologically, patches and plaques are characterized by bandlike or lichenoid infiltrates of lymphocytes in the upper dermis, while the dermal infiltrates become more diffuse in the tumor stage. Pautrier’s microabscesses are the highly characteristic feature of MF and consist of clusters of malignant T cells surrounding Langerhans cells in the epidermis [127,128,129]. On the other hand, SS is the leukemic form of disease that is characterized by erythroderma, generalized lymphadenopathy, and neoplastic T cells in the skin, lymph nodes, and peripheral blood [127,128]. Recently, CD158/KIR3DL2 has been identified as a useful diagnostic surface marker of Sézary cells [130].

The skin-homing effector/memory T cells are known to express CLA and CCR4 [1]. Consistently, the malignant T cells of MF/SS are characterized as CLA^+^CCR4^+^CD4^+^ T cells, while skin lesions express high levels of CCL17 and CCL22 [131]. Furthermore, consistent with the expression of CCR4, malignant CD4^+^ T cells in most cases of MF/SS exhibit a Th2 phenotype with enhanced production of IL-4 and IL-5 [132]. Malignant T cells of MF/SS may also adopt a Treg phenotype with FoxP3 expression and secretion of IL-10 and TGF-β [133]. The Treg-like functions of tumor cells may explain the increased susceptibility of MF and SS patients to opportunistic infections [134].

Besides CCR4, malignant T cells from MF may also express CCR6, CCR7 and CCR10 [135]. Furthermore, in the early stages of MF, malignant T cells may express CXCR3 and CXCR4 at high percentages [136,137]. In the advanced stages of MF, on the other hand, malignant T cells upregulate CCR7, which promotes lymphatic entry of malignant T cells [128,136]. On the other hand, the malignant T cells of SS universally co-express CCR7 and L-selectin as well as the differentiation marker CD27, a phenotype consistent with central memory T cells [34,35]. Of note, central memory T cells recirculate between the blood and SLOs, and a fraction may also migrate to peripheral tissues such as the skin. Thus, the central memory phenotype of malignant T cells in SS well accounts for the clinical features of SS: leukemic cells in circulation, generalized lymphadenopathy, and diffuse erythroderma [138]. Furthermore, SS cells produce CXCL13, which is well-detectable in skin lesions and lymph nodes, and is also present at high concentrations in the blood of SS patients [139]. Since the addition of CXCL13 to CCL19 or CCL21 enhances cell migration via CCR7, production of CXCL13 by SS cells may further enhance their lymph node homing [139]. Collectively, it is now considered that MF is a malignancy of skin resident Tem whereas SS is a malignancy of Tcm [140].

We have also shown that Fra-2 and JunD are highly expressed in CTCL skin lesions together with CCR4 [141]. Furthermore, silencing of Fra-2 or JunD in CTCL cell lines led to the suppression of cell growth and downregulation of CCR4 expression, supporting the oncogenic and CCR4-inducing roles of Fra-2/JunD in CTCLs as in ATLL [141]. In addition, the retinoid X-receptor specific retinoid Bexarotene, which is known to be beneficial to the resolution of cutaneous disease of SS patients, has been shown to induce apoptosis of malignant T cells in SS and also to reduce CCR4 expression with an associated decrease in chemotaxis to CCL17 [142].

## 5. Development and Clinical Application of Mogamulizumab

### 5.1. Development of Mogamulizumab

The Japanese pharmaceutical company Kyowa Hakko (now Kyowa Hakko Kirin) has developed a technology termed Potelligent, which involves the glyco-engineering of a monoclonal antibody to enhance its antibody-dependent cellular cytotoxicity (ADCC). The original observation was that monoclonal IgG_1_ antibodies with low fucose contents had more than a 50-fold higher ADCC activity than the high fucose content counterparts using human peripheral blood mononuclear cells (PBMCs) as effector cells [143]. The company also generated a mouse monoclonal IgG_1_ anti-CCR4 termed KM2160, which was shown to be useful for specific staining of cell surface CCR4 [38]. Since the expression of CCR4 at high levels on malignant T cells provided an attractive therapeutic target for ATLL and CTCLs [95,131], the company first generated a chimeric and defucosylated anti-CCR4 termed KM2760 from KM2160 and confirmed that KM2760 exhibited a much higher ADCC against CCR4-expressing T cell lines in vitro and higher antitumor activities in transplanted mouse models than the highly fucosylated but otherwise identical KM3060 [144]. These promising results led the company to generate a fully humanized anti-CCR4 termed KW-0761. In a preclinical study, KW-0761 showed potent antitumor activity against primary ATLL cells in vitro and in transplanted mice [145]. It was also confirmed that the antitumor activity of KW-0761 was mainly mediated by natural killer (NK) cells present in autologous PBMCs [145].

### 5.2. Clinical Application of Mogamulizumab in ATLL

A phase 1 dose escalating study in patients with relapsed ATLL and peripheral T-cell lymphomas (PTCLs) was performed and reported in 2006. KW-0761 was well-tolerated at all doses (0.01 to 1.0mg/kg) and achieved objected responses in 5 out of 15 evaluable patients irrespective of dose: two complete and three partial responses [146]. The subsequent multicenter phase 2 study for patients with relapsed ATLL was reported in 2012. KW-0761 was given at a dose of 1.0 mg/kg once a week for 8 weeks. Out of 26 evaluable patients, 13 (50%) had objective responses, including 8 complete responses [147]. The most common adverse events were infusion reactions (89%) and skin rashes (63%), which were manageable and reversible in all cases [147]. Lymphopenia was also frequently observed (96%). Upon these favorable results, the Japanese PMDA approved KW-0761 now termed Mogamulizumab in March 2012 for the treatment of patients with replaced ATLL. Post-marketing studies have confirmed the efficacy and adverse reactions observed in the phase 2 study [148,149]. Thus, Mogamulizumab is now regarded as the first-line therapeutic agent for the treatment of relapsed ATLL in Japan. Of note, Mogamulizumab has also shown a better efficacy in ATLL patients with the C-terminal gain-of-function mutations of CCR4 [150].

A phase 2 study of Mogamulizumab in patients with relapsed PTCLs and CTCLs was also conducted in Japan and reported in 2014 with favorable results and an acceptable toxicity profile [151]. Furthermore, a multicenter randomized phase 2 study was conducted for newly diagnosed aggressive ATLL to examine the efficacy and safety of the addition of Mogamulizumab to the current intensified chemotherapy regimen [152]. Although the combination therapy achieved a slightly higher response rate than the chemotherapy alone, it also showed a less favorable safety profile [152]. It thus remains to be seen whether Mogamulizumab is more beneficial to newly diagnosed ATLL than the current chemotherapy.

### 5.3. Clinical Application of Mogamulizumab in CTCLs

The clinical studies of Mogamulizumab in CTCLs were conducted in the US. In a phase 1/2 study, Mogamulizumab efficiently reduced levels of CCR4^+^ malignant T cells and also CCR4^+^ Tregs in CTCL patients [153]. In another phase 1/2 study, the efficacy of Mogamulizumab in 41 pretreated CTCL patients was evaluated [154]. No dose-limiting toxicity was observed and the maximum tolerated dose was not reached in phase 1. In the phase 2 study, patients were given Mogamulizumab at a dose of 1.0 mg/kg once a week for 4 weeks followed by infusion every 2 weeks until disease progression. Among 38 evaluable patients, the overall response rate was 36.8%: 47.1% in SS (*n* = 17) and 28.6% in MF (*n* = 21). Eighteen of 19 patients (94.7%) with blood involvement had a response in the blood, including 11 complete responses [154]. The frequent adverse events of mostly grade 1/2 were nausea, chills, headache, and infusion-related reaction [154]. In a phase 3 randomized study involving 372 patients, Mogamulizumab demonstrated significant improvement in progression-free survival (PFS) and overall response rate (ORR) compared with Vorinostat, a histone deacetylase inhibitor approved for the treatment of CTCLs [155]: 7.6 months vs. 3.1 months in PFS; 28% vs. 5% in ORR [156,157]. Based on these favorable results, the US Food and Drug Administration (FDA) approved Mogamulizumab in August 2018 for the treatment of adult patients with relapsed or refractory MF or SS after at least one prior systemic therapy [156,157].

### 5.4. Clinical Trial of Mogamulizumab in HAM/TSP

HAM/TSP is a progressive neurodegenerative disease that is characterized by chronic inflammation in the spinal cord and accompanying myelopathic symptoms [93,94]. HTLV-1-infected CCR4^+^CD4^+^ T cells are abundantly present in the cerebrospinal fluids (CSFs) and spinal cord lesions [158]. Furthermore, HTLV-1-infected CCR4^+^CD4^+^ T cells have been found to be converted into Th1-like cells through Tax-induced expression of Tbet and thus express CXCR3 and produce IFN-γ. The production of IFN-γ in turn induces the production of the CXCR3 ligands such as CXCL9 and CXCL10 in the spinal cord tissue cells including astrocytes, leading to further infiltration of HTLV-1-infected CCR4^+^CD4^+^ T cells co-expressing CXCR3 into the spinal cord through a positive feedback mechanism [158,159]. Thus, it is considered to be therapeutic to reduce HTLV-1-infected T cells in patients with HAM/TSP.

A phase 1/2a study of Mogamulizumab was conducted involving 21 patients with glucocorticoid-refractory HAM/TSP in Japan [160]. Mogamulizumab with a maximum dose of 0.3 mg/kg was well-tolerated and the most frequent side effects of mostly low grades were skin rash, lymphopenia, and leukopenia. Mogamulizumab promptly reduced the proviral loads and CCR4^+^CD4^+^ T cells as well as CADM1^+^CD4^+^ T cells in the peripheral blood. The levels of CXCL10 and neopterin in CSF were also reduced. Clinically, a reduction in spasticity and a decrease in motor disability were noted in 79% and 32% of patients, respectively. Thus, Mogamulizumab has also shown a therapeutic efficacy in HAM/TSP and warrants further study [160].

## 6. Adverse Events by Mogamulizumab

A recent meta-analysis involving 14 eligible clinical trials has assessed the safety and efficacy of Mogamulizumab alone or in combination with other drugs [161]. A total of 1290 patients with ATLL, PTCLs, CTCLs, and solid tumors were enrolled in these trials. With regard to the efficacy, approximately 43% of participants reached complete or partial responses by Mogamulizumab monotherapy. For patients treated with Mogamulizumab alone, the most common grade ≥3 adverse events were lymphopenia, neutropenia and rash. When patients were treated with a combined therapy of Mogamulizumab and other drugs, the most common grade ≥3 adverse events were lymphopenia. Since lymphopenia was observed in all doses of Mogamulizumab and all Mogamulizumab-related therapies, it is likely to be caused by the pharmacologic effect of Mogamulizumab [161].

As noted above, skin-related adverse events such as erythema multiforme were also frequently seen in ATLL patients treated with Mogamulizumab [147]. Furthermore, patients with favorable responses tended to have higher grades of skin rash [162]. Thus, the skin reactions are now regarded as an indicator of favorable response [162]. The skin adverse events are usually manageable but may require systemic steroid treatment. Furthermore, fatal cases of Stevens-Johnson syndrome have been reported [163,164]. The mechanisms of skin reactions are not entirely clear but likely to be related to autoimmune skin responses unleased by Mogamulizumab-induced Treg depletion [163,165].

Virtually all peripheral blood CD4^+^CD25^high^FoxP3^+^ Treg cells with potent regulatory activity express CCR4 at high levels [53]. In addition, 80% of Tregs express CLA and 73% express CCR6 [53]. Thus, the majority of Treg cells have the skin-homing capacity. In mice, the accumulation of Tregs in the skin and lung airways was shown to be impaired in the absence of CCR4 [166]. Furthermore, mice with a loss of CCR4 in the Treg cell compartment developed lymphocyte infiltration and severe inflammatory disease in the skin and lungs [166]. These findings support the notion that Treg cells play a vital role in the homeostasis of the skin and lungs. Thus, Treg cell depletion by Mogamulizumab may severely disturb the skin homeostasis. Indeed, immunohistochemical studies demonstrated depletion of Treg cells and prominent infiltration of CD8^+^ T cells in Mogamulizumab-induced skin rashes [163,164]. Autoantibodies recognizing keratinocytes and melanocytes were also demonstrated in the sera of patients suffering from Mogamulizumab-induced skin rashes [167]. Furthermore, the presence of autoantibodies in the epidermis was demonstrated in all biopsy specimens of Mogamulizumab-induced skin rashes but not in control specimens from ATLL skin lesions [167]. It has also been reported that the depletion of CCR4^+^ T cells by Mogamulizumab leads to the homeostatic proliferation of CD8^+^ T cells consisting of predominantly newly emerged clones particularly in patients with skin-related adverse events. Therefore, some of the newly emerged CD8^+^ T cell clones may be responsible for the development of autoimmune skin reactions [168]. Mogamulizumab also induces skin rashes in MF and SS patients, although it may be sometimes difficult to differentiate drug-induced skin rashes from the original skin disease [169]. Development of vitiligo, an autoimmune manifestation, was reported in three patients with SS treated with Mogamulizumab, most probably through the depletion of Treg cells [170]. Collectively, the clinical application of Mogamulizumab may have revealed the vital importance of Treg cells in homeostasis of the skin [171].

Other reported severe adverse events associated with Mogamulizumab treatment include reactivation of hepatitis B virus (HBV) [172,173,174], occurrence of brain EBV-positive diffuse large cell B cell lymphoma (EBV^+^ DLBCL) [175,176], and cytomegalovirus (CM) infections [174,177]. These events are all known to be associated with immunocompromised hosts. Thus, Mogamulizumab-induced lymphopenia and leukopenia may be responsible for these adverse events [161].

## 7. Mogamulizumab and Allo-HSCT

Although Mogamulizumab has been proven to have an impressive therapeutic efficacy on ATLL and also on CTCLs, it does not provide a cure. The disease eventually recurs and becomes refractory to Mogamulizumab. This acquired resistance appears to be mostly due to the selection against CCR4 expression in original malignant clones [178,179]. Thus, allo-HSCT still provides only chances for a cure in patients with aggressive ATLL and CTCLs [91,180,181,182]. However, only a small fraction of patients with ATLL can make it to allo-HSCT because of the highly refractory nature of ATLL to chemotherapy and the advanced age of most patients. Furthermore, even after allo-HSCT, only one-third of patients reach the cure condition. Acute graft-vs-host disease (GVHD) is the major limitation of allo-HSCT and causes significant morbidity and mortality in transplanted patients [91,180,181,182]. In GVHD, donor naïve CD4^+^ T cells recognize allo-antigens on host antigen-presenting cells and differentiate into various effector T cells, which infiltrate host organs by using chemokines and chemokine receptors and initiate alloantigen-reactive immune responses [183,184]. Given the impressive clinical efficacy of Mogamulizumab in ATLL patients, often attaining complete remission, it was expected that Mogamulizumab would increase the chances of ATLL patients being able to undergo allo-HSCT [91]. However, it was soon discovered that patients pretreated with Mogamulizumab often experienced severe GVHD. It is now concluded that Mogamulizumab administration in the pre-transplantation setting promotes the occurrence of severe steroid-resistant GVHD, which leads to increases in non-relapse mortality and overall mortality [91,185,186,187]. Similar results have been reported in CTCL patients pretreated with Mogamulizumab prior to allo-HSCT [187,188]. Again, the mechanism of severe GVHD is likely to be due to the depletion of donor-derived Treg cells by residual Mogamulizumab still present in transplanted patients. However, cases who were successfully treated by the combination of Mogamulizumab and allo-HSCT were also reported [189].

## 8. Immune Checkpoint Inhibitors and Application of Mogamulizumab in Solid Tumors

### 8.1. Immune Checkpoint Inhibitors

Cancer cells can be recognized by the host immune system through the expression of cancer antigens such as cancer differentiation antigens, cancer testis antigens, and cancer neoantigens. In particular, cancer neoantigens are generated as the consequence of genetic damage accumulated during cancer progression and provide ideal immunological targets for host CD4^+^ and CD8^+^ T cells [190,191]. Thus, the host immune system can potentially eliminate autologous tumor cells. However, cancer cells are also known to be modified by selection through complex interplays with the host immune system. The process is called cancer immunoediting, which proceeds through three phases: elimination, equilibrium, and escape [192,193]. In the escape phase, tumor cells are able to evade host immune attacks by reducing antigenicity/immunogenicity as well as by generating an immunosuppressive tumor microenvironment, mainly through the attraction of immunosuppressive cell populations such as Treg cells [194,195]. Thus, in cancer immunotherapy, it is of prime importance to revive hitherto suppressed host immune responses to tumor cells. In this context, the blockade of immune checkpoint pathways and depletion of tumor-infiltrating Treg cells may provide highly promising general approaches to enhance host immune response to cancer.

Immune checkpoints represent a plethora of intrinsic inhibitory pathways of the immune system, which are crucial for maintaining self-tolerance and minimizing immunological collateral tissue damage [196,197,198]. Thus, the inhibitors of immune checkpoints may unleash the hitherto suppressed host immune responses to autologous tumors. Indeed, the recent clinical application of immune checkpoint inhibitors such as anti-programmed cell death protein-1 (PD-1) (Pembrolizumab, Nivolumab) and anti-cytotoxic T-lymphocyte-associated protein-4 (CTLA4) (Ipilimumab) has been quite successful in certain types of cancer such as malignant melanoma, non-small cell lung carcinoma, renal cell carcinoma, and bladder cancer [196,197,198]. Furthermore, unprecedented durability lasting years is noted as characteristic of response to the immune checkpoint inhibitors. However, the immune checkpoint inhibitors have also shown serious drawbacks because of the frequent induction of immune-related adverse events, especially in the skin, gastrointestinal system, liver and endocrine system, mostly probably by unleashing subclinical autoimmune responses to autoantigen [199]. Furthermore, the immune checkpoint inhibitors are effective only in a minor fraction of patients (10~25%) even in approved types of cancer [196,197].

The therapeutic resistance to immune checkpoint inhibitors can be classified as primary resistance when patients do not respond at all or secondary resistance (acquired resistance) when patients have a period of initial response followed by progression of disease [200]. As for the primary resistance, there are no definitive biomarkers to predict patient responses to immune checkpoint inhibitors. However, high-level expression of PD-L1 in tumor cells and high tumor mutation burden (TMB) are possible predictors for patient response to anti-PD-1/PD-L1 therapy [201,202,203]. Furthermore, T cell-inflamed “hot tumors” tend to respond better to the therapy than non-T cell inflamed “cold tumors” [202,204]. As for the acquired resistance, a number of possible mechanisms are now proposed [198,200]. However, if the poor response is due to the paucity of host immune cells attacking autologous tumors, it may be overcome by priming host immune cells by suitable cancer vaccines prior to the treatment with immune checkpoint inhibitors [205,206]. The development of safe and effective CTL-inducing adjuvants is also important for the successful clinical application of cancer vaccines [207,208,209]. Another possible reason for poor responses to immune checkpoint inhibitors may be the presence of potent suppressor cells such as Treg cells and myeloid suppressor cells in the tumor microenvironment, which can suppress the activity of host effector cells to autologous cancer cells even in the presence of immune checkpoint inhibitors [210,211,212,213].

### 8.2. Immune Checkpoint Inhibitors and ATLL, CTCLs

In the case of ATLL, it was reported that both neoplastic and normal CD4^+^ T cells in the peripheral blood of patients expressed PD-1 at increased levels compared with CD4^+^ T cells in the blood of normal healthy controls [214]. In 29 skin biopsy samples, PD-1 expression in neoplastic cells was found to be from 0% to 90% with the median values of 50% [215]. Furthermore, patients with a high PD-1 expression tended to have a shorter median survival time than those with a low PD-1 expression (18.2 months vs. 26.0 months) [215]. Primary ATLL cells were also shown to express PD-L1 in a fraction of patients (21.7%). Immunostaining of PD-L1 in ATLL biopsy samples (*n* = 135) has further revealed three groups of patients: one group (7.4%) had clear PD-L1 expression in ATLL cells; the second group (58.5%) showed minimal expression of PD-L1 in ATLL cells but abundant expression of PD-L1 in stromal cells; the third group (34.1%) did not express PD-L1 in any cells [216]. ATLL cases with PD-L1 expression in tumor cells had inferior overall survival compared with ATLL cases with null PD-L1 expression or stromal PD-L1 expression with the median survival time of 7.5, 10.2 and 18.6 months, respectively [216]. A further comprehensive immunostaining study of ATLL biopsy samples (*n* = 69) for immune checkpoint molecules has shown that expression of PD-1, OX40, galectin-9, and PD-L1 on neoplastic cells is nearly mutually exclusive, suggesting the use of distinct immune checkpoint pathways in individual ATLL cases [217]. Moreover, stromal expression of PD-L1, OX40L, and T cell immunoglobulin mucin-3 (Tim-3) has been shown to be significantly associated with better overall survival [217]. Of note, the 3′-untranslated region of the PD-L1 gene is frequently truncated in various human cancer types including ATLL (27%), resulting in overexpression of PD-L1 through a marked elevation of aberrant PD-L1 transcripts [218].

Taken together, these findings suggest that immune checkpoint pathways including the PD-1/PD-L1 axis play important roles in immune evasion of ATLL. Accordingly, a phase 2 trial of Nivolumab (anti-PD-1) was initiated for patients with ATLL in the US. However, the trial had to be stopped after a single infusion of Nivolumab in three patients, one with chronic, one with smoldering, and one with acute but stable disease ATLL, because of rapid progression of the disease in all three patients [219]. It was confirmed that the expansion of original clones was responsible for the disease progression. Thus, the trial may have revealed an unexpected tumor-suppressive role of the PD-1/PD-L1 axis in ATLL [220]. Induction of hyperprogressive disease (HPD) by immune checkpoint inhibitors has also been reported in solid tumors [221]. However, it has been commented that a similar phase 2 trial of Nivolumab for patients with aggressive ATLL in Japan has not observed such a clinical course in eight patients so far treated [222]. The reason for such differences is not clear at present but may be related to the difference in the patient groups: namely, patients with indolent or stable diseases vs. patients with aggressive diseases. At any rate, immune checkpoint inhibitors still remain an important possibility to be tested for ATLL patients. Clinical trials of immune checkpoint inhibitors for patients with CTCLs are also ongoing [223]. For example, a recent phase 2 trial of Pembrolizumab (anti-PD-1) for 24 patients with advanced MF (*n* = 9) or SS (*n* = 15) has demonstrated an overall response rate of 38% with two complete responses and seven partial responses [224].

### 8.3. Effect of Mogamulizumab on Solid Tumors

It has been shown that infiltration of a large number of Tregs in the tumor microenvironment is associated with poor prognosis [210,225]. It has also been shown that CCR4-expressing Treg cells are recruited into tumor tissues by CCL22 that is produced in the tumor microenvironment by tumor cells and tumor-associated macrophages [226,227,228,229,230,231]. In preclinical and clinical studies, depletion of Treg cells by anti-CCR4 or CCR4 antagonists has been shown to augment antitumor immunity [232,233,234,235,236,237,238]. The therapeutic efficacy of Mogamulizumab on ATLL and CTCLs is also considered to be partly due to depletion of immunosuppressive Treg cells. Indeed, Treg cells in the blood and skin lesions of ATLL patients were shown to be efficiently depleted by Mogamulizumab treatment [163,165]. Thus, Mogamulizumab may also be beneficial to patients with solid tumors through the depletion of immunosuppressive Treg cells [239]. In a phase 1a study involving 10 patients with CCR4-negative advanced or recurrent solid cancer, Mogamulizumab in a dose range from 0.1 mg/kg to 1.0 mg/kg was well tolerated, and four out of 10 patients showed stable disease during treatment and long-term survival [234]. Even at 0.1 mg/kg, Mogamulizumab efficiently depleted Treg cells in PBMCs. Th2 cells and Th17 cells were also significantly reduced, while Th1 and CD8^+^ T cells were less affected [234]. Thus, it may be reasonable to expect that a combined treatment with Mogamulizumab and one of the immune checkpoint inhibitors would synergistically augment therapeutic efficacy. A phase 1 study was thus conducted for the safety and efficacy of Nivolumab (Anti-PD1) in combination with Mogamulizumab in patients with advanced solid tumors: six patients in the dose-escalation study and 90 patients in the expansion study with Mogamulizumab at 1.0 mg/kg. The analysis of twelve paired biopsy samples for tumor-infiltrating T cells revealed reduction of effector Treg cells and increase of CD8^+^ T cells in most patients. Confirmed objective responses were observed in patients with several tumor types such as hepatocellular carcinoma (27%) and non-small cell lung cancer (20%). The most frequent adverse events included rash (39%) and maculopapular rash (20%). One Stevens-Johnson syndrome and three type 1 diabetes mellitus were observed. The profile of treatment-related adverse events was not substantially different from that seen with each monotherapy. Thus, the combination of Nivolumab and Mogamulizumab has provided an acceptable safety profile and antitumor activity. However, no clear relationships were observed between the depletion of Treg cells in peripheral blood or tumor tissues and objective tumor responses [240]. Another phase 1 study was conducted with the combination of Mogamulizumab and Durvalumab (anti-PD-L1) or Tremelimumab (anti-CTLA4) [241]. Tolerability was acceptable with no new or unexpected toxicities. Again, Mogamulizumab at 3.0 mg/kg resulted in almost complete depletion of peripheral blood effector Treg cells and reduction of intra-tumoral Treg cells in the majority of patients. There was, however, no clear correlation between clinical responses and reduction of Treg cells. Furthermore, the efficacy of Durvalumab or Tremelimumab was not appreciably enhanced by the addition of Mogamulizumab in patients with advanced solid tumors [241]. Thus, trials of Mogamulizumab in solid tumors have so far failed to provide expected therapeutic effects and warrant further study.

## 9. Concluding Remarks

The advent of Mogamulizumab has greatly improved the prognosis of ATLL patients. A recent multicenter prospective observational study involving 101 patients with ATLL have revealed that the overall response rate, median progression-free survival and overall survival are 65%, 7.4 months and 16.0 months, respectively, with 44 patients attaining complete responses [242]. However, there is also a substantial fraction of patients whose responses to Mogamulizumab are poor. One reason for low response to Mogamulizumab is obviously the low or negative surface expression of CCR4 on malignant T cells. In one study, it was reported that CCR4 was positive in 48/52 (92%) of ATL cases [243]. The fact that the C-terminal truncation of CCR4, which leads to the high constitutive surface expression of CCR4, correlates with the superior efficacy of Mogamulizumab supports this notion [150,244]. The efficacy of Mogamulizumab is also dependent on ADCC that is mostly mediated by NK cells [143,145]. Thus, the levels of NK cell activity in individual patients, which are often compromised by previous chemotherapies, also determine the effectiveness of Mogamulizumab. As noted above, patients who have skin rashes after Mogamulizumab treatment tend to have better therapeutic responses [162]. While skin-infiltrating ATLL cells may have some intrinsic Treg functions [102,245], it is also likely that Mogamulizumab-induced Treg depletion is partly responsible for skin rashes. Thus, the occurrence of skin rashes may prove the presence of strong NK cell activity, which can attack malignant T cells. In this context, continuous intravenous infusion of recombinant IL-15 has been shown to impressively expand NK cells and CD8^+^ T cells as well in the blood and thus may be beneficial to patients who have low NK cell activity prior to Mogamulizumab treatment [246]. Furthermore, depletion of Treg cells by Mogamulizumab may also unleash hitherto suppressed host immune responses against ATLL [233,242]. The same considerations are more or less true for the treatment of CTCLs with Mogamulizumab.

The phase studies of Mogamulizumab alone or in combination with one of the immune checkpoint inhibitors in patients with solid tumors has shown that Mogamulizumab efficiently reduces effector Treg cells in peripheral blood and the tumor microenvironment [234,240,241]. Again, skin rashes were the most frequent adverse events in patients treated with Mogamulizumab. However, Mogamulizumab has not synergistically enhanced the efficacy of immune checkpoint inhibitors in patients with solid tumors so far [240,241]. One reason for such a poor effect of Treg depletion by Mogamulizumab may be related to concomitant depletion of other CCR4-expressing effector T cells such as Th2, Th17 and Th22 (Table 2). In particular, Th17 cells may have an important role in anti-tumor immunity [247,248]. Furthermore, because of the flexibility of T cell differentiation programs [31,32], substantial fractions of Th1 and CD8^+^ T cells also express CCR4 [249,250]. Indeed, lymphopenia of high grades were commonly observed in patients treated with Mogamulizumab [146,147,161]. Of note, in the original phase 1 study of Mogamulizumab for ATLL and PTCL patients, significant clinical responses were observed even at the dose of 0.01 mg/kg [146]. Thus, it may be possible to fine tune the dose of Mogamulizumab, which may still effectively deplete Treg cells but mostly spare other CCR4-expressing immune cell populations in patients with solid tumors. The dose consideration may also be important for ATLL and CTCLs to reduce adverse events and to improve the outcome of allo-HSCT with Mogamulizumab pretreatment [91]. The excessive alloantigen-reactive immune responses after allo-HSCT may also be curtailed by blocking chemokine receptors such as CXCR3 of donor Th1 and CD8^+^ T cells (Table 2).

Collectively, it is hoped that future studies will further optimize the protocols of Mogamulizumab treatment for patients with ATLL, CTCLs, HAM/TSP, and solid tumors to maximize its benefits and minimize its adverse effects.

## Figures and Tables

**Table 1:** Immune chemokines.

Receptor	Chr	Ligand	Chr	Receptor-Expressing Cell Type
CCR4	3p24	CCL17, CCL22	16q13	Th2, Th17, Th22, Treg, Skin-T, Lung-T, Plt
CCR6	6q27	CCL20	2q36.3	Th17, Th22, Treg, Skin-T, Intestine-T, iDC, B
CCR7	17q12–1.2	CCL19, CCL21	9p13.3	Naïve T, Tcm, aDC, B
CCR8	3p22	CCL1	17q11.2	Th2, Skin-T, Treg
CCR9	3p21.3	CCL25	19p13.2	Intestine-T, IgA-ASC
CCR10	17q21.1–21.3	CCL28	5p12	Skin-T, Th22, IgA-ASC
CXCR3	Xq13	CXCL9, CXCL10, CXCL11	4q21.1	Th1, CD8+ T
CXCR4	2q21	CXCL12	10q11.1	B, Naïve T, Memory T, DC, Plt
CXCR5	11q23.3	CXCL13	4q21.1	Tfh, B
CXCR6	3p21	CXCL16	17p13	Th1, NK, NKT
CX3CR1	3p21.3	CX3CL1	16q13	CD8+ T, NK, Mo/Ma
XCR1	3p21.3	XCL1	1q24.2	cDC1

Abbreviations: Chr, chromosome; Th, T helper; Treg, regulatory T; Skin-T, skin-homing T cell; Lung-T, lung-homing T; Plt, platelet; Intestine-T, intestine-homing T cell; iDC, immature dendritic cell; B, B cell; Tcm, central memory T; aDC, activated dendritic cell; IgA-ASC, IgA-antibody secreting cell; CTL, cytotoxic T lymphocyte; DC, dendritic cell; Tfh, T follicular helper; NK, natural killer cell; NKT, natural killer T cell; Mo/Ma: monocyte/macrophage; cDC1, conventional dendritic cell type 1.

**Table 2:** The major T cell subsets and chemokine receptors.

T Cell Subset	Inducing Cytokine	Master Regulator	Signature Cytokine	Major Chemokine Receptor
Th1	IL-12, IFN-γ	T-bet	IFN-γ, IL-2, TNF-β	CXCR3, CCR5
Th2	IL-4	GATA3	IL-4, IL-5, IL-13	CCR4, CCR8, CCR3
Th17	TGF-β, IL-6, IL-21	RORγ	IL-17, IL-22	CCR6, CCR4
Th22	TNF-α, IL-6	AHR	IL-22, TNF-α, IL-13	CCR10, CCR6, CCR4
Tfh	IL-6, IL-21	Bcl-6	IL-4, IL-17, IFN-γ	CXCR5
Treg	TGF-β, IL-2	FoxP3	IL-10, TGF-β	CCR4, CCR6, CCR8

Abbreviations: Th, helper T; Tfh, follicular helper T; Treg, regulatory T; IL, interleukin; IFN, interferon; TGF, transforming growth factor; TNF, tumor necrosis factor; T-bet, T-box expressed in T cells; GATA3, GATA-binding transcription factor 3; RORγ, RAR-related orphan receptor γ; AHR, Aryl hydrocarbon receptor; Bcl-6, B-cell lymphoma 6 protein; FoxP3, Forkhead box P3.
